# The Role of News Consumption and Trust in Public Health Leadership in Shaping COVID-19 Knowledge and Prejudice

**DOI:** 10.3389/fpsyg.2020.560828

**Published:** 2020-10-22

**Authors:** Lindsay Y. Dhanani, Berkeley Franz

**Affiliations:** ^1^Department of Psychology, Ohio University, Athens, OH, United States; ^2^Department of Social Medicine, Heritage College of Osteopathic Medicine, Ohio University, Athens, OH, United States

**Keywords:** COVID-19, trust, media, news, health promotion

## Abstract

The novelty of COVID-19 has created unique challenges to successful public health efforts because it has required the public to quickly learn and formulate knowledge and attitudes about the virus as information becomes available. The need to stay apprised of new information has also created a critical role for mass media and public institutions in shaping the public’s knowledge of, attitudes about, and responses to the unfolding pandemic. In this study, we examine how media consumption and reliance on specific institutions for information shapes three critical outcomes associated with public health epidemics: the accumulation of knowledge and the endorsement of misinformation about COVID-19, and prejudicial responses to the virus. We surveyed 1,141 adults residing across the United States in March 2020. Using multivariate regression and *t*-tests, we found that participants had greater knowledge, were less likely to endorse misinformation, and reported less bias toward Asian Americans when they had higher trust in the CDC and lower trust in President Trump. Reliance on certain news formats and sources was also associated with knowledge, misinformation, and prejudice. Our findings suggest that trust and news consumption can pose critical barriers to health literacy and foster negative prejudicial responses that further undermine public health efforts surrounding the COVID-19 pandemic.

## Introduction

SARS-CoV-2 and the corresponding disease in humans, COVID-19, were first identified in the late months of 2019. As the virus has spread and deaths have accumulated, countries across the globe have responded with substantial public health campaigns to contain the growing pandemic. The novelty of the virus, however, has posed numerous and continued challenges to successfully responding to the unfolding pandemic. In addition to requiring the scientific community to rapidly generate insight into the characteristics of the virus, how it spreads, and how to best prevent and treat it, it also required the dissemination of that knowledge to the public who were quickly formulating new attitudes and beliefs about the virus. Indeed, the public’s knowledge of and response to COVID-19 is arguably the most important component of a successful public health campaign because it is only through widespread adherence to evidence-based practices that we can meaningfully reduce the spread of the virus and its widespread public health, economic, and social consequences.

Over the course of the last few months, different media sources and institutions have taken varied approaches in the ways they have framed messages about COVID-19 to the public. While some media sources have focused on transmitting evidence-based information about COVID-19, others have used tactics such as downplaying the seriousness of the virus, perpetuating conspiracy theories and other misinformation, and scapegoating by placing the blame for the continued outbreak on China (e.g., Chiu, 2020). These differing messages have not only created deep fissures among the public, but have also resulted in harmful behaviors such as refusing to comply with recommended practices to stem transmission (Mervosh et al., 2020), violence toward those who try to enforce such practices (MacFarquhar, 2020), and even rising prejudice toward people of East Asian descent who have become blamed for COVID-19 (Ruiz et al., 2020).

Given the polarization that has emerged regarding COVID-19 and its impact on the public’s response to the unfolding pandemic, we believe it is important to better understand the role of mass media in shaping the public’s knowledge and attitudes related to COVID-19. More specifically, we examine how patterns of media consumption, and trust in key institutions that are issuing guidance to the public, affect the degree to which people hold accurate information about COVID-19, endorse common misinformation about COVID-19, and express stigma toward Asian Americans. The current study focuses on these specific outcomes due to their centrality to public health amid the COVID-19 outbreak. That is, knowledge, misinformation, and prejudice are key determinants of the degree of harm COVID-19 can inflict on physical and social well-being. In the following sections, we first describe the important role mass media and other informational sources can play in shaping attitudes and perceptions of social life. We then explicate how mass media consumption and trust in institutions central to public health can influence knowledge, misinformation, and prejudice in response to COVID-19.

### The Role of Mass Media in Shaping Knowledge and Beliefs

Generally, the media messages the public are exposed to can be pivotal in shaping their perceptions and responses to health crises and other social issues (Randolph and Viswanath, 2004; Anderson, 2009; Sugimoto et al., 2013; Schmidt et al., 2018). The effects of media can be understood from the perspective of social cognition, which broadly refers to the ways people gain, process, store, and apply social information (Fiske and Taylor, 1991). Theoretical models of social cognition vary in their tenets but share the common prediction that such processes often involve attending to and relying on limited and sometimes biased information (Wyer and Radvansky, 1999). Media is one social agent that can produce such biases in social information through communicating and drawing attention to specific knowledge, ideas, values, norms, and behaviors (Shrum, 2002), perhaps at the exclusion of others.

One specific way in which media coverage has the ability to significantly shape public opinion is through framing an issue or topic to suggest what aspects of the issue are most salient (Nelson et al., 1997). Indeed, research on social cognition has found that people do not review all relevant evidence when formulating judgements (Fiske and Taylor, 1991) and media framing can influence what subset of information is determined to be most relevant or sufficient to draw a conclusion (Shrum, 2002). Media also plays a central role in informing the public’s decisions through influencing the accessibility of information available when formulating judgments (Wyer and Radvansky, 1999). That is, people tend to rely on the information they are most readily able to recall, and the media can influence accessibility through repeated messaging that reinforces chosen aspects of a topic (Happer and Philo, 2013). Finally, media can also create sociocultural pressure to conform with the values, norms, or behaviors transmitted by the media content (Barlett et al., 2008; Grabe et al., 2008).

In support of the influential role of mass media, media has been linked to a number of social attitudes, behaviors, and health-related beliefs. As some examples, media consumption has been connected to body image concerns and body satisfaction (Barlett et al., 2008; Grabe et al., 2008), beliefs about climate change (Anderson, 2009), attitudes toward vaccinations (Schmidt et al., 2018), and health literacy (Hayes et al., 2007). There is also early evidence that media may be influencing knowledge and beliefs about COVID-19, which we turn to in the next section.

### Media, Institutions, and Covid-19 Knowledge and Misinformation

The role of mass media in constructing social realities is of particular importance for COVID-19 because, as described above, the novelty of the virus has required the public to formulate new ideas and attitudes about the virus, which have taken shape in the context of the media messages one has been exposed to. Correspondingly, the varied approaches to disseminating information about COVID-19 to the public, coupled with the deep divides in the sources the public relies on for information, have the potential to influence how knowledge and attitudes about the virus have developed. First, media sources and institutions have differed considerably in their chosen framing of the virus which impacts what information will become disseminated to viewers. Framing techniques have included emphasizing the pandemic’s threat to public health, focusing on discussions of civil liberties (Ingraham, 2020), or stressing the economic toll of the virus (Hilton, 2020). If a media outlet chooses to emphasize civil liberties, for example, they may present viewers with information about rights, personal freedom, and how uncomfortable it is to wear a mask at the exclusion of information about the benefits of masks, thus influencing how informed viewers are about this critical health-protective behavior.

Further, people have developed strong preferences for certain media formats and sources in the United States (Mitchell and Oliphant, 2020; Zitner and Chinni, 2020). These preferences are formed by the tendency for people to seek out (Rodriguez et al., 2017) and more favorably evaluate (a process referred to as “motivated skepticism”) (Ditto and Lopez, 1992) information that supports their personal and political motives. Importantly, these divisions are not limited to traditional sources of media but have also extended to U.S. institutions which have, at times, been in disagreement about key information related to the spread, severity, duration, and prevention of COVID-19. In particular, messaging from the President and White House staff has often been at odds with messaging from public health organizations, such as the Centers for Disease Control (CDC), and the degree to which each of these institutions is trusted and relied upon may change the information one is exposed to.

This has further been compounded by the notable absence of the CDC, the nation’s health protection agency, at national press briefings. This organization has historically played a central role in providing important health information to the public, especially during epidemics (Greenfield-Boyce, 2020; Sun, 2020). Their absence may contribute to the existence of echo chambers wherein Americans are exposed to information that is consistent with their political views, even though some of that information may lack an evidence base. Together, this creates a significant barrier to cultivating an informed public because people are then only exposed to the information their trusted sources choose to distribute or emphasize.

Outside of raising concerns about the extent to which the public is exposed to relevant knowledge about COVID-19, there have also been particular fears about the spread of misinformation, or incorrect knowledge, related to COVID-19. Examples of misinformation related to COVID-19 have ranged from inaccurate information about the origin of the virus (e.g., that it was intentionally created and released) to incorrect beliefs about the severity or mortality of the virus. It also appears that this misinformation has reached a large audience, with three out of 10 Americans believing COVID-19 was created in a lab and a majority of Americans agreeing that news coverage is exaggerating risks related to the virus (Mitchell and Oliphant, 2020). This misinformation also carries important consequences, such as when sources downplay the risk of the virus, leading the public to underestimate the harm or the need to take precautions.

We posit media consumption is also likely connected to exposure to and endorsement of misinformation about COVID-19. Previous research on other critical lapses in public trust related to infectious disease, such as with vaccine hesitancy, has examined how and why misinformation persists despite the availability of evidence downplaying inaccurate claims (Lewandowsky et al., 2012). Researchers have argued that the repetition of false claims can make it more difficult to refute this information as can evidence that threatens one’s worldview (Lewandowsky et al., 2012). This again underscores the importance of the media messaging one consumes which can present and continually reinforce false claims. In particular, the growth of the internet and social media have the potential to amplify the dissemination of misinformation by facilitating the easy and rapid release of non-credible information.

### Media, Institutions, and COVID-Prejudice

One final potential outcome of polarized media use observed amid the COVID-19 pandemic is the rising prejudice and discrimination targeting people of east Asian descent (Ruiz et al., 2020). We argue the growing negative sentiments toward Asians must be considered in our evaluations of the effectiveness of the public health response to COVID-19 because experiences of discrimination can doubly disadvantage Asian Americans such that they must contend with the threat of the virus while also enduring racial backlash which can further erode their health and wellbeing (Gee et al., 2007). Additionally, increased prejudice has also generally increased social tensions that impede unified responses to combating the virus.

Though there are enduring tendencies to associate threats for disease with outgroup members (e.g., Faulkner et al., 2004; Navarrete and Fessler, 2006), messaging from the media and key U.S. institutions can also contribute to the observed increases in prejudice toward people of Asian descent in response to COVID-19. Notable examples include President Trump, White House officials, and popular media outlets referring to the virus as the “Chinese virus,” the “Wuhan virus,” or other names that link the virus to China and, by extension, people from that region (Rogers et al., 2020). Similarly, some news sources and officials have devoted considerable time to criticizing Chinese cultural practices or otherwise blaming China for the outbreak (Chiu, 2020). Numerous criticisms have been raised regarding this type of framing because of its potential to fuel prejudice and encourage discriminatory behaviors (Hoppe, 2018). The social cognition perspective summarized above aligns with these criticisms, underscoring how framing and repeated messaging that stigmatizes China can inform attitudes and social judgments. Therefore, we propose that news consumption and trust in institutions will also be related to expressions of COVID-19-specific prejudice toward Asians.

### The Current Study

Trying to combat a global pandemic against the backdrop of inconsistent messaging about the spread, treatment, and symptoms of infectious diseases increases public health risks (Dhillon and Daniel Kelly, 2015). This is because perhaps one of the best remedies for slowing or mitigating the spread of the virus is a well-informed public who trust public health organizations and are willing to implement evidence-based precautions. Without this, people may not be equipped to make informed decisions to protect themselves and others, thus undermining efforts to slow the spread of infection. In addition, individuals may be at further harm if they not only lack the information necessary to engage in health protective behaviors, but endorse misinformation or engage in discriminatory behavior that threatens the well-being of social groups associated with an infectious disease. The aim of this study, accordingly, is to better understand how media consumption and trust in government and public health leadership relates to different facets of knowledge related to COVID-19, including the endorsement of misinformation, and the expression of prejudice toward Asian Americans among individuals living in the United States. More specifically, we examine the news formats and sources people use, and trust in President Trump and the CDC, as predictors of knowledge and attitudes related to COVID-19.

This study makes a novel contribution by applying psychological theories to explain how information and misinformation are transmitted, and prejudice strengthened, through media use during the COVID-19 epidemic. This is critical to understand because exposure to media may affect the accumulation of different forms of knowledge, the ability to identify and ignore misinformation, and decisions to stigmatize outgroups, all of which are central to public health campaigns. In other words, understanding how informational sources vary in their support of health literacy and stigma reduction is critical to an effective public health response to this unfolding pandemic and can inform future public health efforts by directing them toward the media formats and sources they most need to target in campaigns to improve knowledge and attitudes. This study further illuminates the social processes that shape prejudicial reactions to public health crises by identifying how existing preferences for information consumption can give rise to stigmatized perceptions of minority groups.

## Materials and Methods

### Participants

Data were collected from a national sample of 1,141 adults residing in the United States. Participants were recruited to complete a survey on their knowledge and attitudes about the coronavirus outbreak using Qualtrics panels, which is a third-party online participant recruitment service. Qualtrics Panels recruits participants from a pool of potential respondents who have agreed to participate in online market research. This approach to data collection was advantageous for this study because it allowed us to recruit a diverse set of geographically dispersed participants. There were a total of 1,346 participants who were recruited from Qualtrics Panels and entered the survey. Of these participants, 1,141 met our inclusion criteria, passed our quality control checks, and provided complete data, representing a completion rate of 84.8%. Data collection took place between March 13th and March 18th, 2020, during which time the United States was beginning to implement social distancing practices and many large institutions were beginning to close or modify their practices. The study was approved by the institutional review board at [name redacted] and informed consent was obtained from all participants before survey completion.

Our sample was demographically and regionally diverse. Roughly half of our sample identified as male (52.1%), 46.9% identified as female, and 0.8% identified as third gender or non-binary. Examining racial and ethnic identities, 74.7% of the sample identified as White, 13.3% as Black or African American, 7.5% as Hispanic or Latinx, 5.6% as Asian, 2.9% as American Indian or Alaskan Native, 0.6% as Native Hawaiian or Pacific Islander, and 0.5% as Middle Eastern. The average age of our participants was 44.66 (*SD* = 16.96) and ages ranged from 18 to 99 years old.

The majority of our sample identified as heterosexual (87.4%) and 12.6% identified as gay, lesbian, bisexual, or another sexual identity. The sample also varied on political affiliation, with 43.5% identifying as democrats, 28.9% identifying as independents, and 27.6% identifying as republicans. The most common level of education reported was a Bachelor’s degree (23.7%), followed closely by some college but no degree (23.5%) and a high school diploma or equivalent (22.3%). Another 13.3% held an Associate’s degree, 13.6% held a Master’s, and 3.7% held either a doctoral or professional degree. Finally, our sample included participants from all 50 states and the representation for each state ranged from 0.2% (Wyoming) to 16.4% (California).

### Measures

To test our hypotheses, participants were asked to report their level of trust in governmental and health leadership, the news sources they most relied on for information, their knowledge about various facets of COVID-19, and their attitudes about Asian Americans.

#### Institutional Trust

Trust was measured by asking participants to rate their level of trust, ranging from 0 (*no trust*) to 10 (*complete trust*), in the CDC and President Trump. We chose to focus on these two sources because: (1) the CDC is the leading health organization in the U.S. and a key source for evidence-based information about the pandemic, and (2) President Trump has issued frequent statements about the virus, sometimes in conflict with the CDC and other public health leaders, and thus may have a large influence on the public’s knowledge and attitudes.

### News Consumption

We next assessed the various news sources participants relied on for information in two ways. First, and following the distinctions used by Pew Research Center (Shearer, 2018), participants were asked how frequently they relied on television, news websites, radio, social media, and print newspapers and response options ranged from 1 (*never*) to 5 (*always*). This approach was taken because these various news formats have differing standards and requirements related to the credibility of the information they distribute. Next, to increase the specificity of our assessment of news consumption, we asked participants to indicate whether or not they frequently use the following news sources: CNN, Fox News, Facebook, National Public Radio (NPR), the New York Times, and Twitter. This allowed us to better document specific sources that may contribute to knowledge and prejudice related to COVID-19.

#### COVID-19 Knowledge

Knowledge was assessed using a multifaceted knowledge questionnaire developed for the current study. Items were created by integrating surveys on prior infectious disease outbreaks such as the H1N1 outbreak in 2009 (Di Giuseppe et al., 2008) with websites created by health organizations to inform the public and dispel misinformation about COVID-19 (Maragakis, 2020; World Health Organization, 2020). Further, the measure was constructed to measure four facets of knowledge that are critical to public health responses to COVID-19: knowledge of the spread of the virus (6 items), knowledge of the common symptoms of the virus (8 items), information about treatments for the virus (4 items), and an endorsement of misinformation that was circulating at the time of the survey (3 items). Knowledge on the spread of COVID-19 asked participants to identify the methods that can effectively help prevent spreading the virus, including using hand sanitizer, washing hands with soap and water, using saline rinses, wearing facemasks, using hand dryers, and disinfecting surfaces. The measure of virus symptomology asked participants to identify the symptoms of COVID-19 from a checklist of symptoms that included runny nose, sore throat, body aches, cough, fever, nausea or vomiting, shortness of breath, and fatigue.

The third facet assessed knowledge of the treatments for COVID-19 through items asking whether COVID-19 can be prevented by the pneumonia vaccine, whether a current treatment or vaccine exists, if warmer weather will cure the virus, and when a vaccine is expected to be developed. Finally, misinformation was measured by asking participants to indicate their agreement with false statements that were commonly discussed at the time of the survey (i.e., COVID-19 has a similar mortality rate as the flu; COVID-19 is a manmade virus; and it is dangerous to receive a package from China).

#### Prejudice Toward Asians

We developed a 4-item measure to assess prejudice toward people of Asian descent that has arisen because of COVID-19. On a scale ranging from 1 (*extremely unlikely*) to 5 (*extremely likely*), participants were asked to report their likelihood of interacting with people of Asian descent in a variety of contexts. Specifically, participants were asked if they would order food from a restaurant with primarily Asian employees, sit next to an Asian person on a bus or other public transportation, attempt to limit interactions with Asian customers or coworkers, or intentionally move farther away from an Asian individual while in a public place. These behaviors were the focus of our measure because they align with reports of Asian Americans’ experiences in response to COVID-19. The scale demonstrated adequate reliability (α = 0.78) and results from a one-factor confirmatory factor analysis demonstrated good fit [χ^2^(2) = 21.38, *p* < 0.001, CFI = 0.99, TLI = 0.98, SRMR = 0.02].

##### Control Variables

Our analyses also included a number of control variables. First, we controlled for level of education because it is correlated with health literacy (Van Der Heide et al., 2013) and may also be related to general knowledge of viruses. We further controlled for political affiliation, age, gender, and race given the early evidence that COVID-19 beliefs, knowledge, and responses have been stratified by these demographic variables (Alsan et al., 2020; Jurkowitz and Mitchell, 2020; Tyson, 2020).

### Analyses

Analyses were conducted by testing multivariate regression models that predicted each of the four facets of COVID-19 knowledge and bias toward Asians. For each model, we first regressed knowledge scores onto the control variables (i.e., gender, age, race, education, sexual orientation, and political affiliation). Step 2 then added the independent variables (i.e., trust in the CDC, trust in President Trump, and news consumption) to assess their relationship with different forms of knowledge and bias toward Asians. We finally conducted independent samples *t*-tests to assess the effects of using specific news sources on COVID-19 knowledge and bias toward Asians. These analyses compare mean scores on each of the outcome measures for participants who did and did not use each of the six specific news sources described previously. Analyses were conducted using IBM SPSS (Version 26.0).

## Results

### Descriptive Statistics

Prior to testing our hypotheses, we examined the descriptive statistics and distribution of each of our independent variables (see Supplementary Materials). Rated on a scale of 0 to 10, the average trust rating for the CDC was 7.66 (*SD* = 2.36) and the average trust rating for President Trump was 4.17 (*SD* = 3.69). Examining the news sources respondents relied on, participants reported using television news sources most frequently (*M* = 3.77, *SD* = 1.26), followed by news websites (*M* = 3.38, *SD* = 1.24), social media (*M* = 3.18, *SD* = 1.41), radio (*M* = 2.86, *SD* = 1.26), and print newspapers (*M* = 2.51, *SD* = 1.36). These findings are similar to other reports of the most relied upon news sources (Shearer, 2018).

We next calculated the descriptive statistics for the four measures of COVID-19 knowledge. Participants scored an average of 4.79 (*SD* = 0.94) on the items assessing the transmission of the virus (out of a total possible score of 8) and an average of 5.45 (*SD* = 1.28) on the 8 items assessing knowledge of the symptoms of the virus. Further, the average score for the 4 items assessing treatment knowledge was 3.16 (*SD* = 0.92) and the average score for the three misinformation items was 1.22 (*SD* = 0.87). Overall, the descriptive statistics for the knowledge measures suggest participants held moderately accurate knowledge of COVID-19 but also tended to endorse at least some misinformation. Finally, participants scored an average of 2.45 (*SD* = 1.04) on our measure of prejudice toward people of Asian descent. Further, 475 (41.6%) of our participants endorsed that they were somewhat or extremely likely to engage in at least one of the behaviors included in the measure.

### Predicting Knowledge of COVID-19

We calculated the intercorrelations for all study variables to present the bivariate relationships between the control variables, independent variables, and measures of COVID-19 knowledge (displayed in Table 1). We then examined the relationships between media, trust in institutions, and COVID-19 knowledge by conducting four regression models in which each facet of COVID-19 knowledge was predicted by the control variables in Step 1 and the independent variables in Step 2. Results from these models are shown in Table 2.

**TABLE 1:** Intercorrelations among study variables.

	1	2	3	4	5	6	7	8	9	10	11	12	13	14	15	16	17	18
1. Race																		
2. Female	0.014																	
3. Non-binary	0.025	−0.09*																
4. Republican	−0.21*	–0.06	–0.04															
5. Independent	–0.05	0.00	0.04	−0.39*														
6. Age	−0.26*	–0.05	−0.09*	0.13*	–0.02													
7. Education	–0.01	−0.06*	–0.03	0.06*	−0.12*	0.04												
8. Trust in CDC	–0.05	0.03	0.00	0.00	−0.08*	0.12*	0.05											
9. Trust in Trump	−0.19*	−0.13*	–0.05	0.55*	−0.06*	0.10*	0.04	0.01										
10. News: TV	–0.02	−0.07*	−0.08*	0.03	−0.12*	0.23*	0.07*	0.20*	0.07*									
11. News: Websites	0.01	–0.01	−0.07*	–0.02	−0.11*	−0.09*	0.24*	0.18*	0.03	0.28*								
12. News: Radio	0.06*	−0.12*	–0.03	0.06*	−0.11*	0.00	0.24*	0.06*	0.21*	0.29*	0.36*							
13. News: Social media	0.11*	0.05	0.02	–0.06	−0.11*	−0.37*	0.09*	0.06*	0.03	0.09*	0.36*	0.23*						
14. News: Print	0.03	−0.17*	–0.04	0.05	−0.14*	0.08*	0.23*	0.05	0.13*	0.30*	0.28*	0.45*	0.15*					
15. Misinformation	0.05	–0.02	−0.07*	0.08*	0.02	–0.06	−0.14*	−0.16*	0.13*	0.01	−0.10*	0.01	0.11*	0.01				
16. Treatment knowledge	−0.10*	0.14*	0.01	−0.10*	0.03	0.17*	–0.05	0.15*	−0.25*	0.01	–0.03	−0.18*	−0.16*	−0.16*	−0.23*			
17. Symptoms knowledge	−0.11*	0.18*	–0.03	0.00	0.01	0.11*	−0.08*	0.13*	−0.13*	0.03	–0.01	−0.15*	−0.07*	0.21*	−0.13*	0.29*		
18. Spread knowledge	0.02	0.10*	–0.05	–0.04	0.03	0.05	−0.12*	0.07*	−0.17*	–0.02	−0.09*	−0.22*	−0.07*	−0.26*	–0.03	0.23*	0.31*	
19. Anti-Asian attitudes	0.07*	−0.08*	–0.04	0.09*	−0.07*	−0.06*	0.03	−0.11*	0.20*	0.08*	0.02	0.18*	0.12*	0.19*	0.22*	−0.20*	−0.22*	−0.13*

*N = 1,141; race is coded 1 for White/Caucasian and 2 for racial minorities.* * *p* < 0.05.

**TABLE 2:** Regression analyses predicting knowledge about COVID-19.

	Misinformation			Treatments			Symptoms			Spread		
				
Variable	β	SE	95% CI	β	SE	95% CI	β	SE	95% CI	β	SE	95% CI
**Step 1**												
Race	0.127*	0.062	0.006, 0.249	−0.197**	0.064	−0.323, −0.071	−0.280**	0.090	−0.457, −0.104	0.053	0.067	−0.078, 0.185
Female	–0.064	0.051	−0.165, 0.036	0.262***	0.053	0.157, 0.366	0.459***	0.074	0.313, 0.605	0.180**	0.055	0.072, 0.289
Non-binary	−0.693**	0.262	−1.207, −0.179	0.361	0.272	−0.172, 0.894	–0.031	0.380	−0.777, 0.715	–0.343	0.283	−0.899, 0.212
Republican	0.241***	0.064	0.115, 0.367	−0.296***	0.066	−0.426, −0.166	–0.070	0.093	−0.252, 0.113	–0.043	0.069	−0.179, 0.092
Independent	0.117	0.062	−0.004, 0.239	–0.076	0.064	−0.202, 0.050	–0.028	0.090	−0.204, 0.148	0.034	0.067	−0.097, 0.165
Age	–0.003	0.002	−0.006, 0.000	0.010***	0.002	0.007, 0.013	0.007**	0.002	0.003, 0.012	0.003*	0.002	0.000, 0.007
Education	−0.079***	0.017	−0.111, −0.046	–0.026	0.017	−0.060, 0.008	−0.061*	0.024	−0.108, −0.014	−0.068***	0.018	−0.103, −0.032
*R*^2^ (Δ*R*^2^)			0.206***			0.275***			0.243***			0.171***
**Step 2**												
Race	0.115	0.061	−0.004, 0.234	−0.196**	0.062	−0.317, −0.075	−0.261**	0.088	−0.433, −0.089	0.066	0.065	−0.060, 0.193
Female	–0.038	0.051	−0.138, 0.062	0.175**	0.052	0.073, 0.276	0.341***	0.074	0.197, 0.486	0.071	0.054	−0.035, 0.178
Non-binary	−0.683**	0.256	−1.185, −0.181	0.236	0.260	−0.273, 0.746	–0.141	0.370	−0.868, 0.586	–0.500	0.272	−10.034, 0.034
Republican	0.112	0.076	−0.036, 0.260	0.028	0.077	−0.122, 0.179	0.205	0.109	−0.009, 0.420	0.182*	0.080	0.024, 0.339
Independent	0.090	0.063	−0.033, 0.213	–0.004	0.064	−0.128, 0.121	0.036	0.091	−0.142, 0.214	0.064	0.067	−0.067, 0.195
Age	0.000	0.002	−0.004, 0.003	0.007***	0.002	0.004, 0.011	0.006*	0.002	0.001, 0.011	0.003	0.002	−0.001, 0.006
Education	−0.072***	0.017	−0.105, −0.039	–0.009	0.017	−0.042, 0.025	–0.036	0.025	−0.084, 0.013	–0.031	0.018	−0.066, 0.005
Trust in CDC	−0.054***	0.011	−0.075, −0.033	0.049***	0.011	0.027, 0.070	0.057***	0.016	0.026, 0.088	0.029*	0.012	0.006, 0.052
Trust in Trump	0.024**	0.009	0.008, 0.041	−0.063***	0.009	−0.080, −0.046	−0.050***	0.012	−0.074, −0.026	−0.043***	0.009	−0.061, −0.025
News: TV	0.036	0.022	−0.008, 0.080	0.015	0.023	−0.029, 0.060	0.056	0.032	−0.007, 0.120	0.043	0.024	−0.003, 0.090
News: Websites	−0.095***	0.024	−0.141, −0.049	0.045	0.024	−0.002, 0.092	0.057	0.034	−0.010, 0.125	–0.007	0.025	−0.057, 0.042
News: Radio	0.005	0.024	−0.041, 0.052	−0.056*	0.024	−0.104, −0.009	–0.046	0.034	−.113, 0.022	−0.075**	0.025	−0.124, −0.025
News: Social media	0.104***	0.021	0.064, 0.145	−0.063**	0.021	−0.104, −0.022	–0.016	0.030	−0.075, 0.042	0.005	0.022	−0.038, 0.049
News: Print	0.012	0.021	−0.030, 0.054	−0.066**	0.022	−0.109, −0.024	−0.167***	0.031	−0.228, −0.106	−0.136***	0.023	−0.181, −0.092
*R*^2^ (Δ*R*^2^)			0.318(0.059)***			0.414(0.095)***			0.351(0.064)***			0.344(0.089)***

* p < 0.05, ** p < 0.01, *** p < 0.001.

The first model, which predicted knowledge of transmission, indicated that knowledge was higher among women (*b* = 0.180, *p* < 0.01), older adults (*b* = 0.003, *p* < 0.05), and less educated participants (*b* = −0.068, *p* < 0.001). Further, an examination of the news and trust variables entered in Step 2 showed that, after controlling for race, gender, political affiliation, age, and education, knowledge scores were higher among people with greater trust in the CDC (*b* = 0.029, *p* < 0.05) and lower trust in President Trump (*b* = −0.043, *p* < 0.001). Further, a greater reliance on radio news sources was associated with less accurate knowledge about COVID-19 transmission (*b* = −0.075, *p* < 0.01), as was a greater reliance on print newspapers (*b* = −0.136, *p* < 0.001).

The next model predicted knowledge of the symptoms of COVID-19. Results for the demographic variables showed significant relationships between knowledge and race (*b* = −0.280, *p* < 0.01), being female (*b* = 0.459, *p* < 0.001), age (*b* = 0.007, *p* < 0.01), and education (*b* = −0.061, *p* < 0.05). Results from Step 2 demonstrate that knowledge of COVID-19 symptoms was higher among participants with a greater trust in the CDC (*b* = 0.057, *p* < 0.001) and lower trust in President Trump (*b* = −0.050, *p* < 0.001). The only news format related to knowledge of symptoms was print media, and greater reliance on print news was associated with less symptom-related knowledge (*b* = −0.167, *p* < 0.001).

Results from the third model, which predicted knowledge of COVID-19 treatments, indicated that treatment knowledge was significantly higher among White participants (*b* = −0.197, *p* < 0.01), women (*b* = 0.262, *p* < 0.001), and older participants (*b* = 0.010, *p* < 0.001). Knowledge of treatment was also significantly lower among republicans (*b* = −0.296, *p* < 0.001). Further, trust in the CDC was significantly positively related (*b* = 0.049, *p* < 0.001), and trust in President Trump was significantly negatively related (*b* = −0.063, *p* < 0.001), to knowledge of COVID-19 treatments. Of the news variables, reliance on radio sources (*b* = −0.056, *p* < 0.05), social media (*b* = −0.063, *p* < 0.01), and print sources (*b* = −0.066, *p* < 0.01) were associated with less accurate knowledge of treatments.

The final knowledge model assessed endorsement of misinformation. An examination of the demographic variables showed that misinformation was significantly higher among racial minority participants (*b* = 0.127, *p* < 0.05) and republicans (*b* = 0.241, *p* < 0.001), and significantly lower among non-binary participants (*b* = −0.693, *p* < 0.01) and more educated participants (*b* = −0.079, *p* < 0.001). Participants with more trust in the CDC endorsed significantly less misinformation (*b* = −0.054, *p* < 0.001) whereas participants with more trust in President Trump endorsed greater misinformation (*b* = 0.024, *p* < 0.01). Finally, reliance on news websites was significantly negatively associated with misinformation (*b* = −0.095, *p* < 0.001) and reliance on social media was associated with increased misinformation (*b* = 0.104, *p* < 0.001).

### Predicting Prejudice Toward Asians

Results for the regression model predicting prejudice toward people of Asian descent is shown in Table 3. Of the demographic variables entered in Step 1, significant relationships were found for race (*b* = 0.186, *p* < 0.05), being female (*b* = −0.173, *p* < 0.01), identifying as republican (*b* = 0.232, *p* < 0.01), and age (*b* = −0.004, *p* < 0.05). These findings suggest prejudice was higher for racial/ethnic minorities and republicans and significantly lower for women and younger adults. In Step 2, prejudice was significantly lower among participants with a greater trust in the CDC (*b* = −0.049, *p* < 0.001) and significantly higher among participants with greater trust in President Trump (*b* = 0.052, *p* < 0.001). Prejudice was also significantly lower among participants who relied on news websites (*b* = −0.067, *p* < 0.05) and higher among participants who relied on radio (*b* = 0.066, *p* < 0.05), social media (*b* = 0.057, *p* < 0.05), and print news sources (*b* = 0.100, *p* < 0.001).

**TABLE 3:** Regression analyses predicting negative attitudes toward Asian Americans in response to COVID-19.

Variable	β	SE	95% CI
**Step 1**			
Race	0.186*	0.074	0.040,0.331
Female	−0.173**	0.061	−0.293, −0.053
Non-binary	–0.537	0.313	−1.151,0.077
Republican	0.232**	0.077	0.082,0.383
Independent	–0.049	0.074	−0.194,0.096
Age	−0.004*	0.002	−0.008,0.000
Education	0.011	0.020	−0.028,0.050
*R*^2^ (Δ*R*^2^)			0.172***
**Step 2**			
Female	0.167*	0.072	0.027,0.308
Non-binary	–0.067	0.060	−0.185,0.051
Race	–0.389	0.302	−0.981,0.203
Age	–0.037	0.089	−0.212,0.138
Education	–0.083	0.074	−0.228,0.062
Republican	–0.003	0.002	−0.007,0.001
Independent	–0.012	0.020	−0.051,0.027
Trust in CDC	−0.049***	0.013	−0.074, −0.024
Trust in Trump	0.052***	0.010	0.033,0.072
News: TV	0.038	0.026	−0.014,0.089
News: Websites	−0.067*	0.028	−0.122, −0.013
News: Radio	0.066*	0.028	0.011,0.121
News: Social media	0.057*	0.024	0.009,0.105
News: Print	0.100***	0.025	0.050,0.149
*R*^2^ (Δ*R*^2^)			0.337(0.084)***

* p < 0.05, ** p < 0.01, *** p < 0.001.

### The Effects of Specific News Sources

As described in the “Materials and Methods” section, participants were also asked follow up questions about the specific news sources they frequently use and we examined whether the use of these specific sources is related to COVID-19 knowledge and prejudice. To do so, we calculated *t*-tests to compare people who frequently used each of the sources (i.e., CNN, Fox News, Facebook, Twitter, National Public Radio [NPR], and the New York Times) to people who did not use these sources to determine if consuming each source of media impacted knowledge and prejudice related to COVID-19.

Results, shown in Tables 4, 5, indicate that people who used Fox News scored significantly higher on misinformation (*t* = −4.117, *p* < 0.001) and prejudice (*t* = −2.392, *p* < 0.05), and significantly lower on treatment knowledge (*t* = 2.766, *p* < 0.01) than people who did not use Fox News. Participants who frequently used Facebook similarly scored significantly higher on misinformation (*t* = −3.360, *p* = 0.001) and prejudice (*t* = −3.574, *p* < 0.001), and significantly lower on knowledge of COVID-19 treatment (*t* = 3.900, *p* < 0.001) and symptoms (*t* = 2.387, *p* < 0.05). Participants who frequently used Twitter scored significantly lower on knowledge of treatment (*t* = 2.621, *p* < 0.01) and knowledge of symptoms (*t* = 2.538, *p* < 0.05).

**TABLE 4:** *T*-tests comparing knowledge scores for people who use and do not use specific news sources.

	Treatment				Symptoms		
		
News source	*N*	*M*	*SD*	*T*	*M*	*SD*	*T*
**CNN**							
Use	462	3.17	0.96	–0.382	5.45	1.31	–0.080
Don’t use	679	3.15	0.89		5.45	1.26	
**Facebook**							
Use	409	30.01	0.98	3.900***	5.33	1.40	2.387*
Don’t use	732	3.24	0.88		5.52	1.97	
**Fox News**							
Use	398	30.06	0.88	2.766**	5.45	1.22	0.029
Don’t use	743	3.21	0.94		5.45	1.31	
**NPR**							
Use	137	3.32	0.86	−2.242*	5.53	1.24	–0.822
Don’t use	1,004	3.13	0.93		5.44	1.28	
**New York Times**							
Use	215	3.25	0.90	–1.684	5.46	1.38	–0.090
Don’t use	926	3.13	0.92		5.45	1.25	
**Twitter**							
Use	215	30.00	10.02	2.621**	5.23	1.46	2.822*
Don’t use	926	3.19	0.89		5.50	1.23	

	**Spread**				**Misinformation**		
		
**News source**	***N***	***M***	***SD***	***T***	***M***	***SD***	***T***

**CNN**							
Use	462	4.81	0.95	–0.422	1.16	0.86	20.054*
Don’t use	679	4.78	0.93		1.27	0.88	
**Facebook**							
Use	409	4.77	0.98	0.737	1.34	0.85	−3.360**
Don’t use	732	4.81	0.91		1.16	0.88	
**Fox News**							
Use	398	4.75	0.92	1.240	1.37	0.88	−4.117***
Don’t use	743	4.82	0.94		1.15	0.86	
**NPR**							
Use	137	4.79	0.90	0.065	0.86	0.88	5.256***
Don’t use	1,004	4.79	0.94		1.27	0.86	
**New York Times**							
Use	215	4.79	0.94	0.124	0.99	0.90	4.397***
Don’t use	926	4.79	0.94		1.28	0.86	
**Twitter**							
Use	215	4.72	10.04	1.230	1.26	0.91	–0.674
Don’t use	926	4.81	0.91		1.22	0.86	

* p < 0.05, ** p < 0.01, *** p < 0.001.

**TABLE 5:** *T*-tests comparing prejudice scores for people who use and do not use specific news sources.

	Prejudice			
	
News Source	*N*	*M*	*SD*	*T*
**CNN**				
Use	462	2.42	10.09	0.729
Don’t use	679	2.46	0.99	
**Facebook**				
Use	409	2.59	10.08	−3.574***
Don’t use	732	2.36	10.00	
**Fox News**				
Use	398	2.55	10.03	−2.392*
Don’t use	743	2.39	10.04	
**NPR**				
Use	137	2.26	1.11	20.082*
Don’t use	1,004	2.47	10.02	
**New York Times**				
Use	215	2.25	10.06	30.070**
Don’t use	926	2.49	10.02	
**Twitter**				
Use	215	2.50	1.19	–0.732
Don’t use	926	2.43	10.02	

* p < 0.05, ** p < 0.01, *** p < 0.001.

Further, misinformation scores were significantly lower among participants who frequently use CNN (*t* = 2.054, *p* < 0.05) compared to those who do not as well as among participants who frequently use the New York Times (*t* = 4.397, *p* < 0.001) as compared to those who do not. Prejudice was similarly lower among participants who frequently used the New York Times (*t* = 3.070, *p* < 0.01) as compared to those who did not. Finally, participants who frequently used NPR scored significantly lower on misinformation (*t* = 5.256, *p* < 0.001) and prejudice (*t* = 2.082, *p* < 0.05), and significantly higher on treatment knowledge (*t* = −2.242, *p* < 0.05). Results from our *t*-tests demonstrate the importance of the specific news sources one relies on in determining not only knowledge but also prejudice connected to COVID-19.

## Discussion

In this study, we assessed whether the types and sources of media people consume, and institutions in which people place their trust, are associated with three negative public health consequences: lack of disease-specific knowledge; the endorsement of misinformation related to COVID-19; and prejudice toward Asian Americans in response to COVID-19. Our results provide support for the role of media and institutional trust in determining all three of these important outcomes and provide evidence that media framing shapes the knowledge people accumulate, their ability to identify misinformation, and attitudes related to COVID-19. This findings help identify specific barriers that may prevent more effective and positive public health responses in the United States. In the sections below, we elaborate on each of these findings and their implications.

### Trust in Institutions

The current study demonstrates the importance of the public’s trust in public health and governmental institutions. More specifically, our results suggest that greater trust in the CDC was associated with increased knowledge, less acceptance of misinformation, and lower prejudice toward Asian Americans. This supports the positive role of trust in health organizations, demonstrating that individuals are more likely to hold critical public health information and resist scapegoating if they believe that the leading public health agency is trustworthy. By contrast, greater trust in the President Trump was associated with decreased knowledge and greater endorsement of misinformation. Individuals trusting information from President Trump, who has continued to link the origin of the outbreak to China, also report greater prejudice toward Asian Americans. This suggests that the presence of informational sources that associate infectious diseases with specific racial/ethnic groups may have significant public health impacts beyond the development of health literacy and contributes to the continued debate about the use of China-centric language and naming to describe COVID-19 (Rogers et al., 2020).

These findings challenge and extend previous findings on the role of trust in determining health attitudes and behaviors. Namely, extant research examining trust has identified *mistrust* in government as a critical barrier to positive health attitudes and behaviors (Jamison et al., 2019; Larson et al., 2018; Whetten et al., 2006). In contrast, our findings suggest that trust in governmental leadership can be a hindrance to health literacy when the messages issued by governmental leaders are at odds with those from public health organizations and emerging evidence-based practices. Practically, this finding also highlights that messaging from governmental leaders in the United States may be impeding effective public health responses to COVID-19.

### Media Consumption

In terms of media consumption, we find effects of the news mediums people consume on both knowledge and attitudes related to COVID-19. First, we find that social media use is associated with several negative consequences, including the endorsement of misinformation related to COVID-19, lower knowledge about how the disease is treated, and greater prejudice toward Asian Americans. This may be because social media allows for the easy and widespread distribution of information while also having minimal standards to assess the credibility of such information. This explanation comports with the recent finding that Americans who rely most on social media reported seeing more misinformation about the pandemic than those who rely on other sources (Jurkowitz and Mitchell, 2020).

In addition, there is increasing evidence that information provided on some social media platforms, such as Facebook, is curated by both users and platform algorithms, according to political affiliation and other characteristics (Bakshy et al., 2015). This increases the chances that individuals may consume information that confirms existing views rather than contributes to the accumulation of evidence-based public health information. Reinforcing this even further, social media users may only follow and friend others who share similar ideologies and post similar content which can create a false consensus wherein users may believe that most people share their COVID-19 knowledge, beliefs, and attitudes, thus making their knowledge and beliefs seem more credible. Given the growing reliance on social media as a news source (Shearer, 2018), public health agencies may benefit from targeting information and bias-reduction campaigns at those who rely on forms of social media such as Facebook for information.

Further, individuals using print and radio sources were less common in our study, but these sources were also associated with lower knowledge of COVID-19 and greater stigmatization of Asian Americans. Because these sources are more often utilized by Americans over 65, these platforms may emphasize political perspectives that are more common among this cohort and which systematically downplay COVID-19 risks or emphasize the relationship of the virus to China. Finally, and in support that some media formats can have positive influences during public health crises, individuals using web news sources were less likely to endorse misinformation. These findings overlap with recent Pew Research Center findings that individuals utilizing national news on news websites are likely to closely follow COVID-19 updates which could help account for why the endorsement of misinformation was lower (Jurkowitz and Mitchell, 2020).

Finally, comparisons of people who use specific news sources, such as Fox News, Twitter, and Facebook, showed that this type of news consumption may bolster prejudice and impair knowledge about COVID-19. Use of NPR, by contrast, was associated with more positive knowledge development and lower prejudice. Given the stark divide in media preferences along partisan lines (Mitchell and Oliphant, 2020), it seems that different media sources influence knowledge and attitudes, likely through the way COVID-19 is framed. For example, downplaying the virus or comparing it to the flu, criticizing public health recommendations such as social distancing, or promoting misinformation are some ways that news outlets may play an outsized role in influencing knowledge and attitudes related to COVID-19. Our findings highlight that partisan divides in news consumption materially impact the accumulation of public health knowledge, the ability to identify and discount misinformation, and attitudes toward minority racial/ethnic groups. Public health officials should consider the political divide in media consumption a critical barrier to overcome in the promotion of health literacy related to COVID-19.

### Practical Implications

A successful public health response to any infectious disease epidemic relies on adequate knowledge of the disease itself and a willingness to make personal sacrifices to reduce transmission. The proliferation of new information related to COVID-19, much of which has not been scientifically validated, has created what the World Health Organization calls an “infodemic” (The Lancet Infectious Diseases Editorial Board, 2020). There is an essential need, therefore, to communicate evidence-based information, but public health leaders are facing critical barriers, especially the deep divides both in the messages different news sources and institutions have emphasized amid the pandemic and in the sources the public trusts and relies on for information. The polarization of media usage and trust in both President Trump and the Centers for Disease Control in the United States appears to shape different types of knowledge of the virus and the endorsement of misinformation. Importantly, the public health impact of diverse messaging extends beyond the development of health literacy and is materially affecting the health of Asian Americans who are stigmatized for their association with the virus’ origin in China.

As such, public health should focus specifically on countering misinformation and addressing the different messages that Americans are receiving from various information sources. In addition, public health messages should be framed in a way that is politically neutral as political affiliation seems to shape responsiveness to public health leadership in the United States. In doing so, public health leaders stand to enhance trust in and the widespread dissemination of evidence-based public health recommendations. Public health organizations and officials can also use the findings from this study to direct their public health campaigns to reach people who are most in need. While it may not be possible for such organizations to control and prevent the spread of inaccurate or harmful information via media outlets, they can target the sources of media most detrimental for health literacy (e.g., social media) to provide consumers with accurate information that may help to counteract more negative messaging.

### Limitations and Future Research

Our study has several limitations which are important to note. First, we conducted this study in March of 2020 as the COVID-19 epidemic was still growing in the United States. Because we measured knowledge and beliefs early on in the epidemic, it is possible that our results could change as information becomes more publicly available and as more scientific studies are published. The amount and types of misinformation that have circulated have also grown since the time of our survey and additional work is needed to assess the more complex conspiracy theories and false beliefs the public may now endorse. However, despite these drawbacks, we believe gathering information at the onset of the spread of COVID-19 in the U.S. is informative as behaviors and attitudes in the early weeks will be formative in determining the virus’s severity.

Second, there are also limitations with our methodology that impact the conclusions we can draw from our data, including the reliance on a cross-sectional survey design which does not allow us to determine causality as well as the quantitative nature of our measures. Given the growing complexity of knowledge and attitudes related to COVID-19, future qualitative studies are warranted to explore how media shapes trust in different information sources and different facets of knowledge related to COVID-19. Finally, the amount of variance explained in our models suggests there are also other variables outside of those explored in the current study that may affect the degree to which the public holds accurate knowledge, endorses misinformation, and expresses prejudice toward people of Asian descent. For example, having a personal connection to someone who has gotten COVID-19 may influence knowledge through directly exposing individuals to COVID-19 information and/or motivating these individuals to become more informed to support their friends or family through their illness. Additionally, individual differences such as personality variables and competing belief systems have been connected to beliefs in conspiracy theories (Swami et al., 2010; Newheiser et al., 2011) and may also be related to COVID-19 knowledge and misinformation.

## Conclusion

To summarize, our findings suggest that various news formats and informational sources shape how much individuals know about the virus and whether individuals hold stigma toward Asian Americans who have become associated with the virus. These findings are important because they identify the key role of mass media and public institutions in affecting the accumulation of knowledge necessary to keep the public safe in an infectious disease epidemic and with beliefs that threaten the health and wellbeing of a subsection of Americans. The polarized nature of American media consumption, in particular, creates an environment where individual beliefs are often reinforced rather than challenged. There is a profound need, accordingly, for public health leaders to construct effective messaging related to COVID-19 that is available to all Americans and is politically neutral, and to combat mistrust in key public health agencies tasked with providing critical public health information to the public.

## Data Availability Statement

The raw data supporting the conclusions of this article will be made available by the authors, without undue reservation.

## Ethics Statement

The studies involving human participants were reviewed and approved by the Ohio University Institutional Review Board. Written informed consent was obtained from all participants and was received electronically.

## Author Contributions

LD and BF contributed to the conception and design of the study, oversaw data collection and wrote portions of the manuscript, and edited the manuscript. LD organized the data and performed the statistical analysis. Both authors read and approved the final manuscript.

## Conflict of Interest

The authors declare that the research was conducted in the absence of any commercial or financial relationships that could be construed as a potential conflict of interest.
