# Supplementary material for: The Role of News Consumption and Trust in Public Health Leadership in Shaping COVID-19 Knowledge and Prejudice
Source: Front Psychol. 2020 Oct 22;11:560828. doi: 10.3389/fpsyg.2020.560828 (PMC7642623; doi:10.3389/fpsyg.2020.560828)
Supplement: Supplementary file 1 [file Data_Sheet_1.DOCX]

**Supplemental Material**

Table A1. Means, Standard Deviations, and Normality Indices for Study Variables.

|  | *M* | *SD* | *Skew* | *Kurtosis* |
| --- | --- | --- | --- | --- |
| Race | 1.25 | .44 | 1.14 | -.71 |
| Female | .47 | .50 | .13 | -1.99 |
| Nonbinary | .01 | .10 | 10.05 | 99.18 |
| Republican | .28 | .45 | 1.00 | -1.00 |
| Independent | .29 | .45 | .93 | -1.14 |
| Age | 44.66 | 17.00 | .27 | -.95 |
| Education | 2.96 | 1.54 | .36 | -.73 |
| Trust in CDC | 7.66 | 2.36 | -1.16 | .98 |
| Trust in Trump | 4.17 | 3.69 | .23 | -1.47 |
| News: TV | 3.77 | 1.26 | -.73 | -.54 |
| News: Websites | 3.38 | 1.24 | -.46 | -.69 |
| News: Radio | 2.86 | 1.26 | .07 | -.97 |
| News: Social media | 3.18 | 1.41 | -.23 | -1.22 |
| News: Print | 2.51 | 1.36 | .43 | -1.05 |
| Misinformation | 1.22 | .87 | .15 | -.78 |
| Treatment knowledge | 3.16 | .92 | -.96 | .44 |
| Symptoms knowledge | 5.45 | 1.28 | -.70 | 1.00 |
| Spread knowledge | 4.79 | .94 | -.72 | .61 |
| Anti-Asian attitudes | 2.45 | 1.04 | .23 | -.66 |

Note: *N* = 1,141; race is coded 1 for White/Caucasian and 2 for racial minorities; the standard error for skew was .072 and the standard error was .145.
